# Supplementary material for: Unifying the design space and optimizing linear and nonlinear truss metamaterials by generative modeling
Source: Nat Commun. 2023 Nov 21;14:7563. doi: 10.1038/s41467-023-42068-x (PMC10663604; doi:10.1038/s41467-023-42068-x)
Supplement: Supplementary file 1 — Supplementary Information [file 41467_2023_42068_MOESM1_ESM.pdf]

Unifying the design space of and optimizing linear and nonlinear truss metamaterials by generative modeling

Li Zheng, Konstantinos Karapiperis, Siddhant Kumar, and Dennis M. Kochmann

Supplementary Information

1 Dataset generation

Supplementary Figure 1 shows the details of the generation of a diverse truss lattice dataset. Supplementary Figure 2 shows the node positions and connectivities of the five considered elementary truss structures. While we did not consider specific manufacturability constraints in this work, it is possible to enforce such constraints to narrow down the design space, depending on the requirements of specific applications. For instance, geometric constraints can be imposed to limit the maximum size or number of edges of the truss graph, while printability constraints[1] can be considered by ensuring that every node has at least one supporting node. Such considerations can easily be incorporated into our graph-based parameterization through conditions on the structure's geometry, utilizing the graph representation. Moreover, our framework can be extended to consider structural instability or failure, e.g., by integrating buckling strength[2] or compressive loading curves[3,4] among the target properties towards the inverse design of buckling-resistant structures. Our generative modeling framework based on a graph representation of truss structures can be adapted to incorporate specific design considerations such as manufacturability requirements, making it a flexible framework that can be tailored to meet specific application goals. In addition, one advantage of our approach is that – when identifying an optimal structure – the latent space provides many structures with similar properties (nearby points in the latent space), so that secondary targets can also be implemented by choosing from a selection of potential trusses with different architectures yet similar effective stiffness.

2 Machine learning framework

2.1 Protocols for NN training

Details of the optimized dimensions and hyperparameters (e.g., the number of hidden layers and nodes in each layer, activation functions, learning rates, etc) of the variational autoencoder model and the property predictor are provided in Supplementary Table 1.

	**Encoder** $\mathcal{Q}_\phi(G)$		**Decoder** $\mathcal{P}_\theta(G)$		**Property predictor** $\mathcal{F}_\omega$
	Connectivity $\boldsymbol{A}$	Node positions $\boldsymbol{x}$	Connectivity $\boldsymbol{A}$	Node positions $\boldsymbol{x}$	
Input dimensions	278	27	$d_\mathrm{A}(40)$	$d_\mathrm{x}(40)$	$d(48) = d_\mathrm{A}(40) + d_\mathrm{x}(40) - d_\mathrm{Ax}(32)$
Hidden dimensions	$512, 512, 512, 128$	$640, 640, 640, 512$	$128, 512, 512, 512$	$256, 512, 640, 640$	$400, 800, 1000, 400, 400, 200$
Output dimensions	$d(48) = d_\mathrm{A}(40) + d_\mathrm{x}(40) - d_\mathrm{Ax}(32)$		278	27	9
Activation functions	ReLU		ReLU		ReLU
Optimization algorithm	Adam[5]		Adam[5]		Adam[5]
Learning rate	5×10^{-4}		5×10^{-4}		5×10^{-4}
Batch size	512		512		512
Drop out	none		none		none

Supplementary Table 1. Dimensions and training hyperparameters of the optimized Variational Autoencoder and the property predictor models.

2.2 Overlapping embedding model

In order to accommodate the interdependencies between the structural topology (represented by the adjacency matrix) and node placements (represented by node features), we modify the VAE structure by restricting which part of a latent embedding is used for each task, as illustrated in Supplementary Fig. 4. Let $d_\mathrm{A} - d_\mathrm{Ax}$ be the number of latent dimensions allocated exclusively to topology, $d_\mathrm{x} - d_\mathrm{Ax}$ the number of dimensions exclusively allocated to geometry, and d_Ax the number of dimensions allocated to both. To keep the overall number of trainable parameters fixed, we reduce the outputs of the encoder ($\boldsymbol{\mu}^\mathrm{A}, \log \boldsymbol{\sigma}^\mathrm{A} \in \mathbb{R}^{d_\mathrm{A}}$, and $\boldsymbol{\mu}^\mathrm{x}, \log \boldsymbol{\sigma}^\mathrm{x} \in \mathbb{R}^{d_\mathrm{x}}$) to obtain the final latent

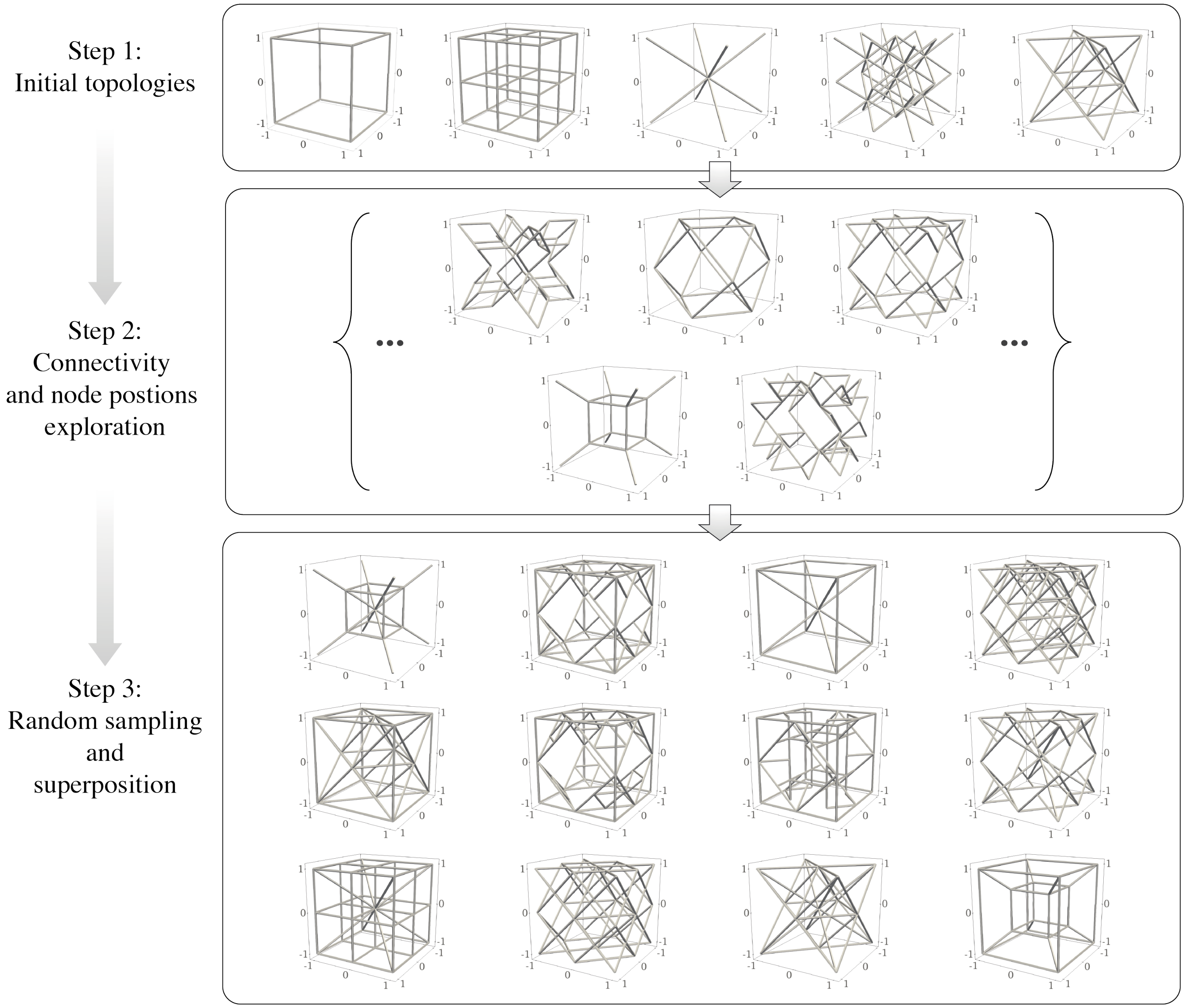

Supplementary Figure 1. Overview of the truss dataset generation. Starting from the five elementary truss lattices shown in Step 1, new structures are created by randomly perturbing both the node positions and connectivities for several iterations. Each geometry is perturbed for 10 iterations to generate a library that contains a wide range of truss structures with several unique topologies, as shown in Step 2. From the established set, two lattices are randomly sampled with repetitions allowed, which are then superimposed according to their matching nodes to yield a more diverse dataset in Step 3.

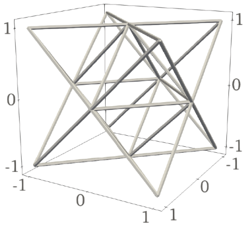

$$\boldsymbol{x} = \begin{bmatrix} 0 & 0 & 1 \\ 0 & 0.5 & 0.5 \\ 0 & 1 & 0 \\ 0.5 & 0 & 0.5 \\ 0.5 & 0.5 & 0 \\ 0.5 & 0.5 & 1 \\ 0.5 & 1 & 0.5 \\ 1 & 0 & 0 \\ 1 & 0.5 & 0.5 \\ 1 & 1 & 1 \end{bmatrix} \quad \boldsymbol{A} = \begin{bmatrix} 1 & 2 \\ 1 & 4 \\ 2 & 3 \\ 4 & 8 \\ 3 & 5 \\ 1 & 6 \\ 3 & 7 \\ 5 & 8 \\ 8 & 9 \\ 6 & 10 \\ 7 & 10 \\ 9 & 10 \end{bmatrix}$$

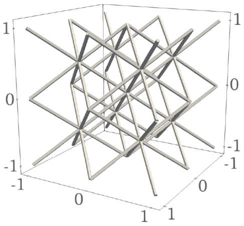

$$\boldsymbol{x} = \begin{bmatrix} 0 & 0 & 0 \\ 0 & 0 & 1 \\ 0 & 1 & 0 \\ 0 & 1 & 1 \\ 0.5 & 0.5 & 0.5 \\ 1 & 0 & 0 \\ 1 & 0 & 1 \\ 1 & 1 & 0 \\ 1 & 1 & 1 \end{bmatrix} \quad \boldsymbol{A} = \begin{bmatrix} 1 & 5 \\ 2 & 5 \\ 3 & 5 \\ 4 & 5 \\ 6 & 5 \\ 7 & 5 \\ 8 & 5 \\ 9 & 5 \end{bmatrix}$$

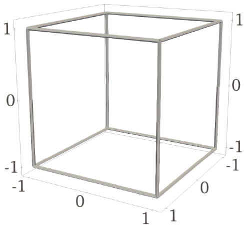

$$\boldsymbol{x} = \begin{bmatrix} 0 & 1 & 1 \\ 1 & 0 & 1 \\ 1 & 1 & 0 \\ 1 & 1 & 1 \end{bmatrix} \quad \boldsymbol{A} = \begin{bmatrix} 1 & 4 \\ 2 & 4 \\ 3 & 4 \end{bmatrix}$$

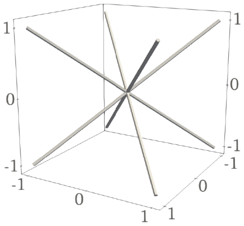

$$\boldsymbol{x} = \begin{bmatrix} 0 & 0 & 0 \\ 0.5 & 0.5 & 0.5 \\ 1 & 1 & 1 \end{bmatrix} \quad \boldsymbol{A} = \begin{bmatrix} 1 & 2 \\ 2 & 3 \end{bmatrix}$$

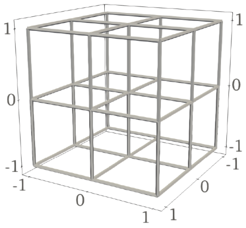

$$\boldsymbol{x} = \begin{bmatrix} 0 & 0 & 0 \\ 0 & 0 & 0.5 \\ 0 & 0 & 1 \\ 0 & 0.5 & 0 \\ 0 & 0.5 & 1 \\ 0 & 1 & 0 \\ 0 & 1 & 0.5 \\ 0 & 1 & 1 \\ 0.5 & 0 & 0 \\ 0.5 & 0 & 1 \\ \ldots & \ldots & \ldots \end{bmatrix} \begin{bmatrix} \ldots & \ldots & \ldots \\ 0.5 & 1 & 0 \\ 0.5 & 1 & 1 \\ 1 & 0 & 0 \\ 1 & 0 & 0.5 \\ 1 & 0 & 1 \\ 1 & 0.5 & 0 \\ 1 & 0.5 & 1 \\ 1 & 1 & 0 \\ 1 & 1 & 0.5 \\ 1 & 1 & 1 \end{bmatrix} \quad \boldsymbol{A} = \begin{bmatrix} 1 & 2 \\ 2 & 3 \\ 1 & 4 \\ 3 & 5 \\ 4 & 6 \\ 6 & 7 \\ 5 & 8 \\ 7 & 8 \\ \ldots & \ldots \end{bmatrix} \begin{bmatrix} \ldots & \ldots \\ 1 & 9 \\ 3 & 10 \\ 11 & 18 \\ 16 & 18 \\ 18 & 19 \\ 12 & 20 \\ 17 & 20 \\ 19 & 20 \end{bmatrix}$$

Supplementary Figure 2. Node positions $\boldsymbol{x}$ and adjacency matrix $\boldsymbol{A}$ of each of the five considered elementary truss unit cells. Each row of the matrix $\boldsymbol{x}$ corresponds to the 3D coordinates of a node in the truss unit cell, while the matrix $\boldsymbol{A}$ defines connectivities between the nodes with given indices in the truss unit cell.

representations $\boldsymbol{\mu}, \log \boldsymbol{\sigma} \in \mathbb{R}^d$ with $d = d_{\mathrm{A}} + d_{\mathrm{x}} - d_{\mathrm{Ax}}$ as

$$\boldsymbol{\mu} = \boldsymbol{\mu}^{\mathrm{A}}_{1:d_{\mathrm{A}}-d_{\mathrm{Ax}}} \oplus \frac{1}{2}\left(\boldsymbol{\mu}^{\mathrm{A}}_{d_{\mathrm{A}}-d_{\mathrm{Ax}}+1:d_{\mathrm{A}}} + \boldsymbol{\mu}^{\mathrm{x}}_{1:d_{\mathrm{Ax}}}\right) \oplus \boldsymbol{\mu}^{\mathrm{x}}_{d_{\mathrm{Ax}+1}:d_{\mathrm{x}}}, \tag{1}$$

$$\log \boldsymbol{\sigma} = \log \boldsymbol{\sigma}^{\mathrm{A}}_{1:d_{\mathrm{A}}-d_{\mathrm{Ax}}} \oplus \frac{1}{2}\left(\log \boldsymbol{\sigma}^{\mathrm{A}}_{d_{\mathrm{A}}-d_{\mathrm{Ax}}+1:d_{\mathrm{A}}} + \log \boldsymbol{\sigma}^{\mathrm{x}}_{1:d_{\mathrm{Ax}}}\right) \oplus \log \boldsymbol{\sigma}^{\mathrm{x}}_{d_{\mathrm{Ax}+1}:d_{\mathrm{x}}}, \tag{2}$$

where $\oplus$ denotes vector concatenation. Our rationale is that the topological and geometrical features of truss lattices are strongly correlated and, therefore, the overlapping dimensions should learn to extract the shared information between different entities, while preserving the topology-specific and geometry-specific information within their respective dimensions of latent representations. Compared to the non-overlapping model, where shared information is stored redundantly, the overlapping model, with its ability to capture joint information of different features, allows us to adjust the importance given to their interdependencies, while maintaining a constant number of training parameters. A comprehensive evaluation of the overlapping and non-overlapping models' performance across various tasks can be found in ref.[6]. The prediction accuracy of the model with different latent space dimensions is shown in Supplementary Table 2. We observe that increasing the shared dimensions allocated to both beyond a certain point does not significantly improve the model's performance. Therefore, we select the latent dimensions $d_{\mathrm{A}} = 8$, $d_{\mathrm{Ax}} = 32$, and $d_{\mathrm{x}} = 8$, which strikes a reasonable balance between capturing essential features of truss structures and maintaining computational efficiency.

By decomposing different factors of variation while preserving the explicit interdependence between factors, the model captures the underlying relationships between these entities more effectively by exploiting the decomposed representations. This decomposition not only enhances the interpretability of the model[7] but also improves its ability to extrapolate beyond the training distribution[8]. Overall, the above characteristics of the latent space make it a meaningful and structured representation of the original discrete design space, which is advantageous for the efficient optimization and generation of novel truss structures with targeted properties.

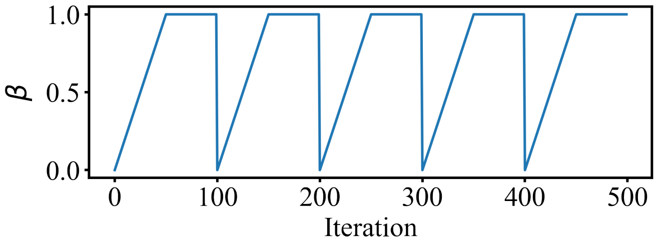

Supplementary Figure 3. Annealing the KL-divergence term weight β with a cyclical schedule. The KL-divergence term weight β starts with 0 at the beginning of training, increases at a fast rate until $\beta = 1$ is reached, and then stays at $\beta = 1$ for subsequent learning iterations. This annealing process is repeated several times to achieve better performance.

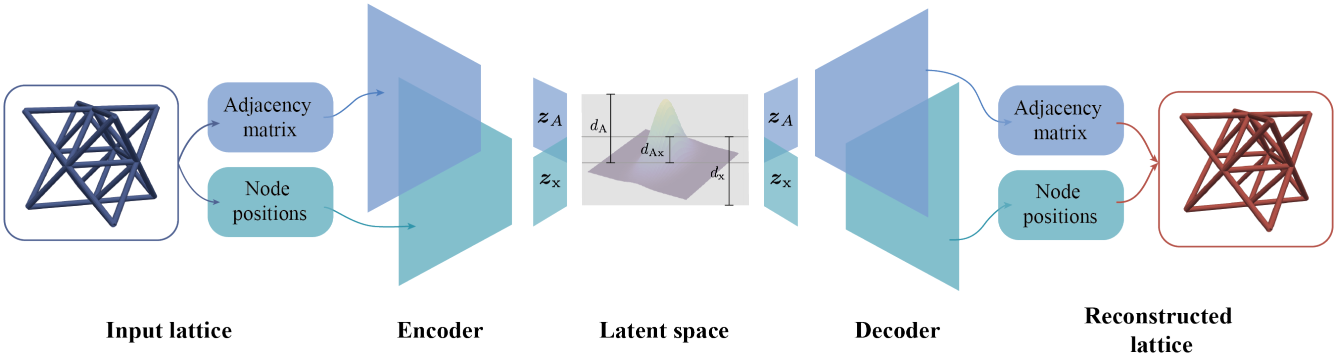

Supplementary Figure 4. Schematic of the overlapping embedding model. $d_{\mathrm{A}} - d_{\mathrm{Ax}}$ is the number of latent dimensions allocated exclusively to topology; $d_{\mathrm{x}} - d_{\mathrm{Ax}}$ is the number of dimensions allocated exclusively to geometry; d_{Ax} is the number of dimensions allocated to both.

2.3 NN model performance

Supplementary Figure 5a and b present the distribution and statistics of the mean and standard deviation of each dimension of the latent space. To illustrate the generative capacities of the VAE model, we randomly sampled latent vectors from the prior d-dimensional Gaussian distribution $\boldsymbol{z} \sim \mathcal{N}(0, \boldsymbol{I})$, where $d = d_{\mathrm{A}} + d_{\mathrm{x}} - d_{\mathrm{Ax}}$. The candidate latent vectors can then be decoded into novel truss structures beyond the training dataset, as shown in detail in Supplementary Fig. 5c. We evaluate the fidelity of the latent space by examining the percentage of randomly sampled points that can be decoded into valid truss structures satisfying the connectivity constraint (as described in Supplementary Note 1). Further, Supplementary Figure 6 lists the reconstruction accuracy of node positions along with representative examples of the reconstructed vs. true truss structures. Supplementary Figure 7 shows the prediction accuracy of all stiffness tensor components for the property predictor $\mathcal{F}_{\omega}$. The prediction accuracies are evaluated on a separate test dataset containing 2000 lattices and their corresponding homogenized stiffness tensor components.

In Supplementary Fig. 8 we further examine the two extreme outliers in Supplementary Fig. 6a-y by comparing

R^2-scores for	$\boldsymbol{A}$	(x, y, z)	$\mathbb{C}_{1111}$	$\mathbb{C}_{1122}$	$\mathbb{C}_{1133}$	$\mathbb{C}_{2222}$	$\mathbb{C}_{2233}$	$\mathbb{C}_{3333}$	$\mathbb{C}_{2323}$	$\mathbb{C}_{3131}$	$\mathbb{C}_{1212}$
$d_{\mathrm{A}} = 28$, $d_{\mathrm{Ax}} = 8$, $d_{\mathrm{x}} = 28$	0.990	0.995	0.985	0.957	0.974	0.976	0.967	0.982	0.970	0.974	0.968
$d_{\mathrm{A}} = 32$, $d_{\mathrm{Ax}} = 16$, $d_{\mathrm{x}} = 32$	0.990	0.996	0.972	0.969	0.962	0.989	0.978	0.932	**0.991**	0.971	0.955
$d_{\mathrm{A}} = 40, d_{\mathrm{Ax}} = 32, d_{\mathrm{x}} = 40$	**0.999**	**0.999**	**0.995**	0.987	**0.989**	**0.995**	**0.990**	**0.995**	0.983	**0.983**	**0.982**
$d_{\mathrm{A}} = 44$, $d_{\mathrm{Ax}} = 40$, $d_{\mathrm{x}} = 44$	0.989	0.994	0.965	**0.990**	0.958	0.977	0.983	0.954	0.969	0.958	0.973

Supplementary Table 2. Comparison of the node positions reconstruction accuracy of the VAE model and the prediction accuracy of the property predictor using different latent dimension $d_{\mathrm{A}}, d_{\mathrm{Ax}}, d_{\mathrm{x}}$. R^2-scores are used as the primary metric.

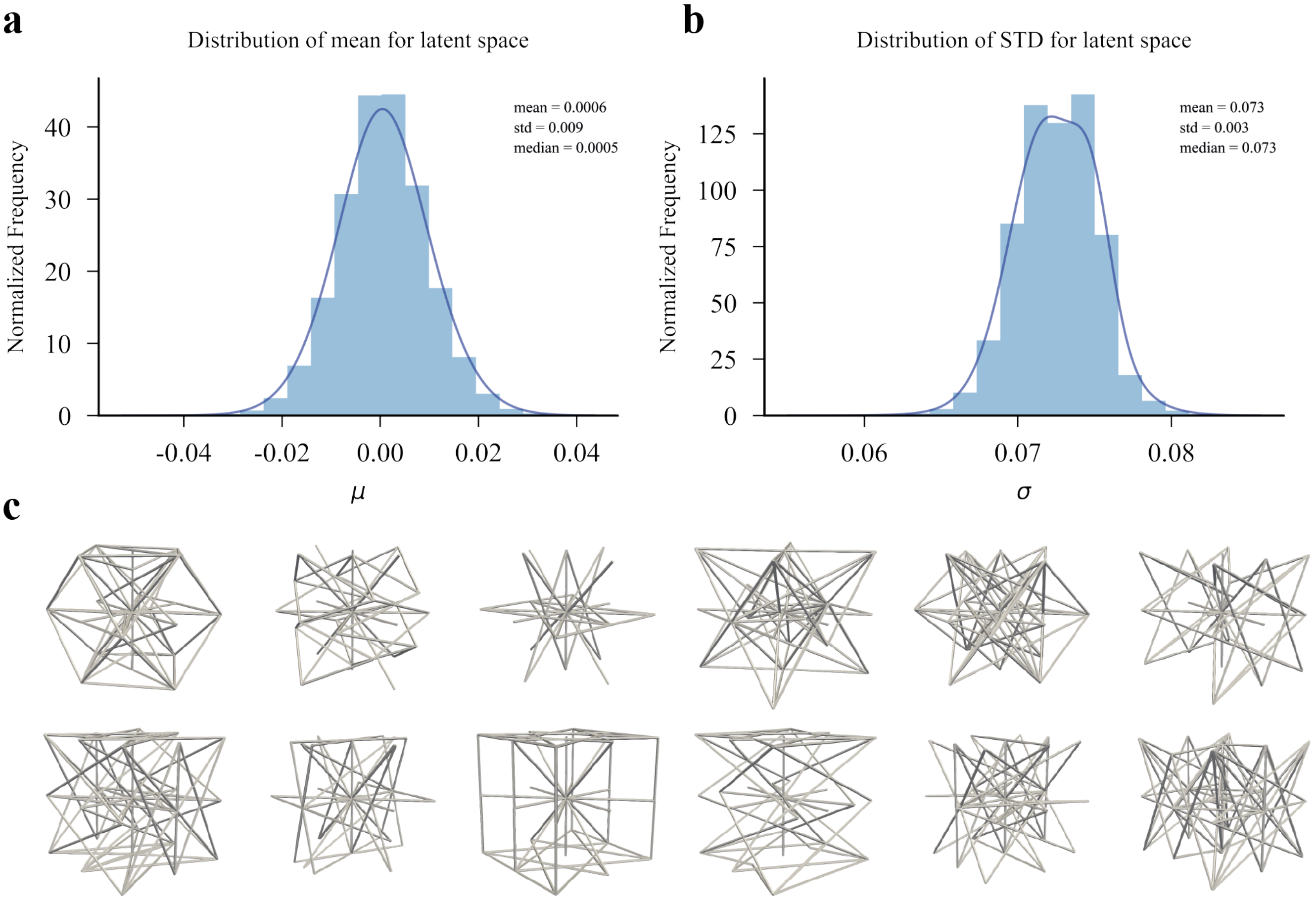

Supplementary Figure 5. Distribution and statistics of the latent space. **(a)** Distribution of the mean of the latent space coordinates. **(b)** Distribution of the standard deviation of the latent space coordinates. **(c)** Representative examples of truss structures generated by decoding from randomly-sampled points from the latent space.

the reconstructed vs. true structure and their corresponding elastic surfaces of the two outliers. We observe that, although there is a significant difference in the reconstructed vs. true node positions of structure (b) in Supplementary Fig. 8a, the difference in their effective structure and properties is minor. In addition, Supplementary Figure 6 shows that the reconstructed node positions correlate well with the ground truth with R^2-values (≈ 0.999) close to unity for each component. Over 99.9% of all instances in the test data had errors within the interval $[-0.048, 0.048]$ for component $y \in [0,1]$, indicating that the overall reconstruction performance is satisfactory. Therefore, we are confident that the VAE obtains a reliable estimation of the intrinsic underlying distribution and provides a sufficiently good reconstruction of various truss structures.

Despite near-perfect overall accuracy, there are several potential reasons for the presence of extreme outliers in the correlation plots. One possible explanation is the trade-off between loss terms in this multi-task learning problem. Specifically, the VAE model is jointly trained with the property predictor as

$$\begin{aligned}\theta,\phi,\omega \leftarrow \underset{\theta,\phi,\omega}{\arg\min}\; & \underbrace{\frac{1}{N}\sum_{n=1}^{N}\left(\left\|\boldsymbol{A}^{(\mathrm{n})}-\boldsymbol{A}^{(\mathrm{n})'}\right\|^2+\left\|\boldsymbol{x}^{(\mathrm{n})}-\boldsymbol{x}^{(\mathrm{n})'}\right\|^2\right)}_{\text{reconstruction loss}}+\underbrace{\frac{1}{N}\sum_{n=1}^{N}\left\|\boldsymbol{S}^{(n)}-\mathcal{F}_\omega[\boldsymbol{\mu}^{(n)}]\right\|^2}_{\text{property prediction loss}} \\ & +\underbrace{\sum_{n=1}^{N}D_{\mathrm{KL}}\left(\mathcal{N}\left(\left[\mu_1^{(n)},\ldots,\mu_d^{(n)}\right]^{\mathsf{T}},\mathrm{diag}\left(\left[{\sigma_1^{(n)}}^2,\ldots,{\sigma_d^{(n)}}^2\right]^{\mathsf{T}}\right)\right)\,\|\,\mathcal{N}(\boldsymbol{0},\boldsymbol{I})\right)}_{\text{Kullback–Leibler divergence}},\end{aligned} \tag{3}$$

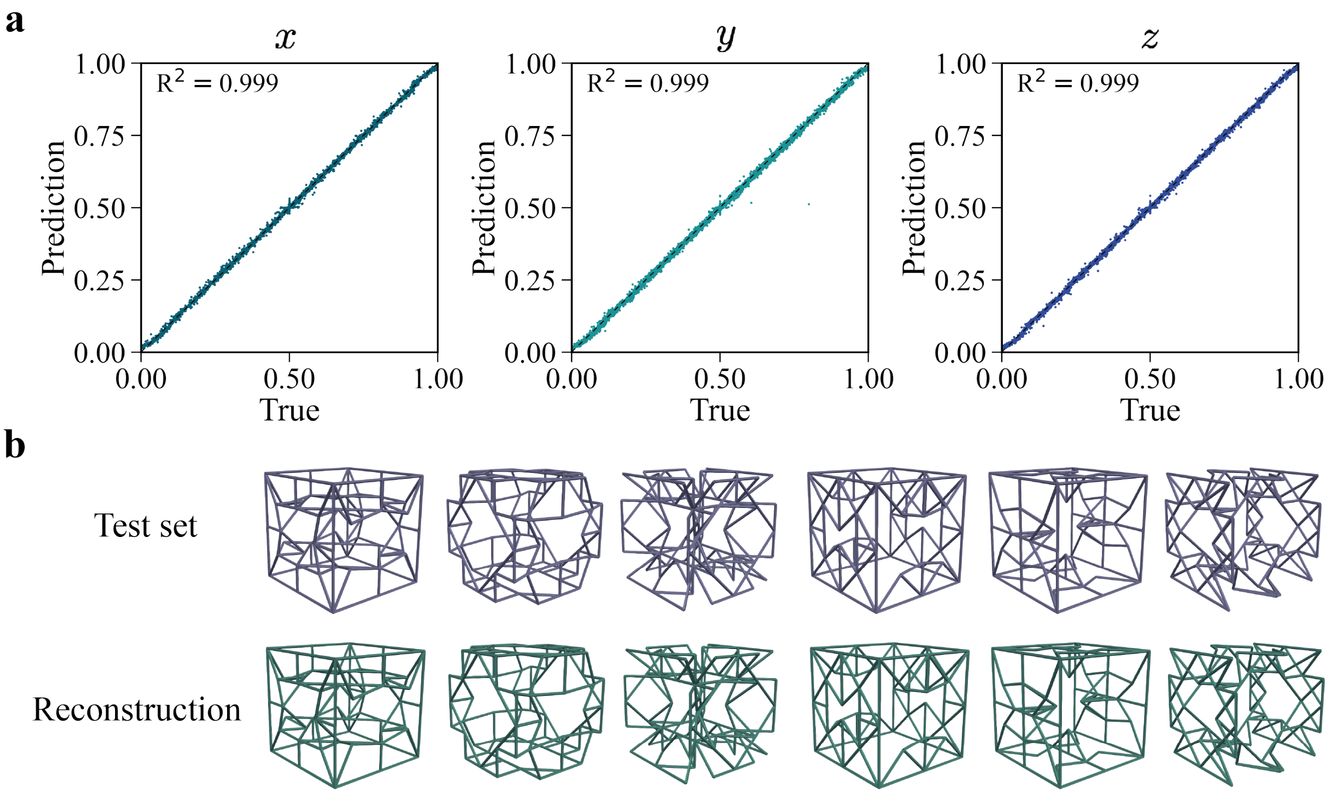

Supplementary Figure 6. Evaluation of the reconstruction accuracy of the variational autoencoder model. (a) Reconstructed vs. true 3D components (x, y, z) of the node positions in the test dataset. The reconstructed node positions were computed using the decoder. Dashed lines represent the ideal lines with zero-intercept and unit-slope; the corresponding R^2-scores are indicated. **(b)** Representative examples of reconstructed vs. true truss structures generated by the variational autoencoder model.

where θ, ϕ, ω are model parameters of the VAE. Minimizing multiple loss functions simultaneously can be challenging because, e.g., the model capacity is limited or some loss terms are in conflict such as the inherent trade-off between the reconstruction robustness and generalization (given by the Kullback-Leibler divergence) in VAEs. A common strategy is to balance the losses as weighted sums of related terms during the multi-objective optimization, whereas the choice of weights will have a strong impact on the performance of the model on different tasks. In this work, we have compared the model performance with several selections of weights and selected the best model based on the property prediction accuracy, because our focus is the downstream task of optimizing truss lattices for target properties.

Alternatively, one can choose the best model according to other criteria depending on actual goals. For example, we modify Supplementary Equation 3 to include a weight $\lambda_{\text{recon}} > 0$ for the reconstruction loss as follows:

$$\theta, \phi, \omega \leftarrow \underset{\theta,\phi,\omega}{\arg\min} \; \underbrace{\lambda_{\text{recon}} \frac{1}{N} \sum_{n=1}^{N} \left(\left\| \boldsymbol{A}^{(\text{n})} - \boldsymbol{A}^{(\text{n})\prime} \right\|^2 + \left\| \boldsymbol{x}^{(\text{n})} - \boldsymbol{x}^{(\text{n})\prime} \right\|^2 \right)}_{\text{reconstruction loss}} + \underbrace{\frac{1}{N} \sum_{n=1}^{N} \left\| \boldsymbol{S}^{(n)} - \mathcal{F}_{\omega}[\boldsymbol{\mu}^{(n)}] \right\|^2}_{\text{property prediction loss}}$$
$$+ \underbrace{\sum_{n=1}^{N} D_{\text{KL}} \left(\mathcal{N} \left(\left[\mu_1^{(n)}, \ldots, \mu_d^{(n)} \right]^{\intercal}, \text{diag} \left(\left[{\sigma_1^{(n)}}^2, \ldots, {\sigma_d^{(n)}}^2 \right]^{\intercal} \right) \right) \parallel \mathcal{N}(\boldsymbol{0}, \boldsymbol{I}) \right)}_{\text{Kullback–Leibler divergence}} . \quad (4)$$

After training the model with a higher weight of the reconstruction loss term ($\lambda_{\text{recon}} = 5$, as opposed to $\lambda_{\text{recon}} = 1$ originally), Supplementary Figure 8b shows that the points are more centralized around the line with unit-slope and zero-intercept with fewer outliers in the correlation plots of reconstructed vs. true node positions (evaluated on

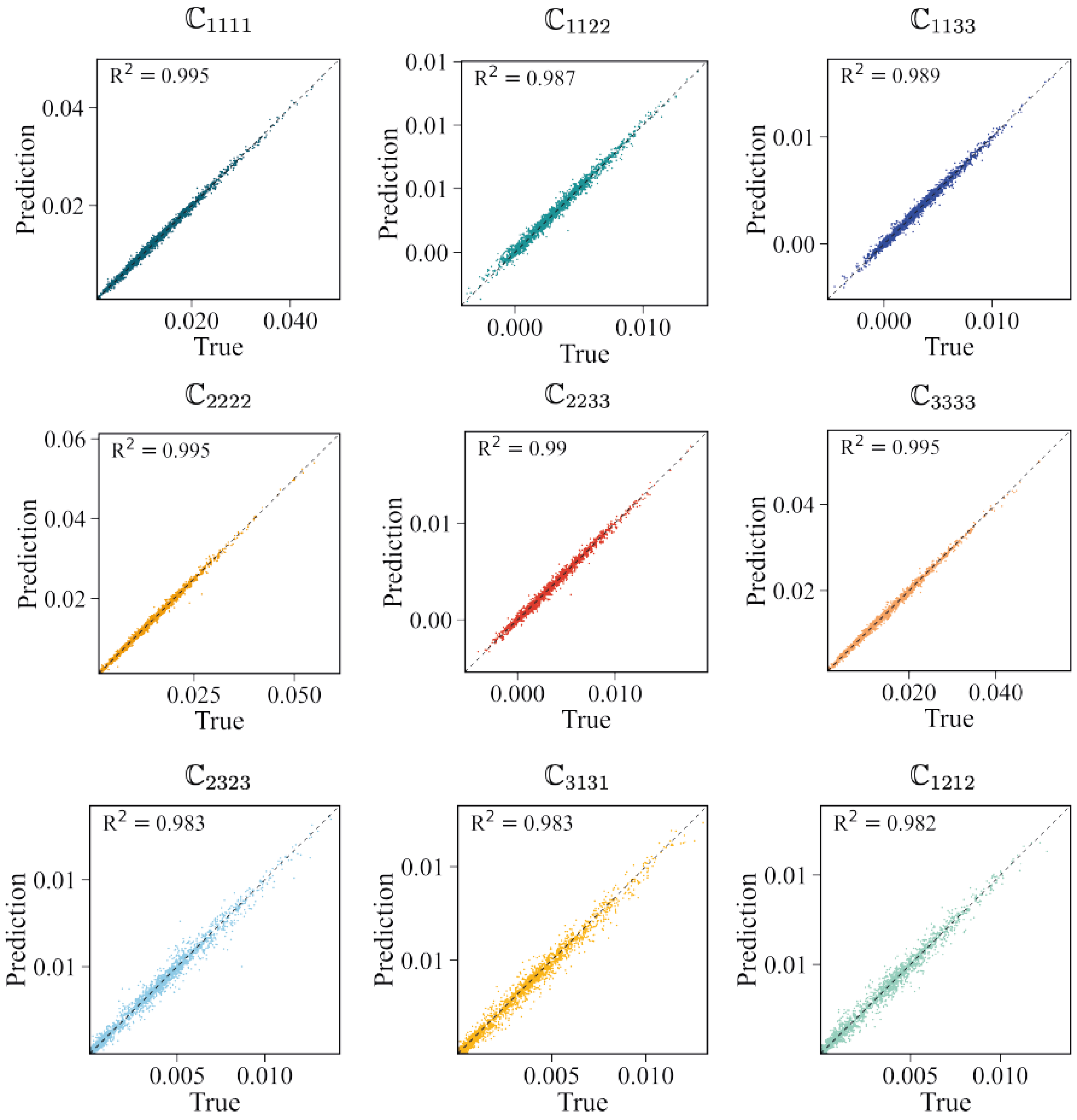

Supplementary Figure 7. Evaluation of the prediction accuracy of the property predictor model. Predicted vs. true components of the stiffness tensor $\mathbb{C}$ in the test dataset. The predicted stiffness is computed using the property predictor $\mathcal{F}_\omega$. All dashed lines represent the ideal line with zero-intercept and unit-slope; the corresponding R^2-scores are indicated.

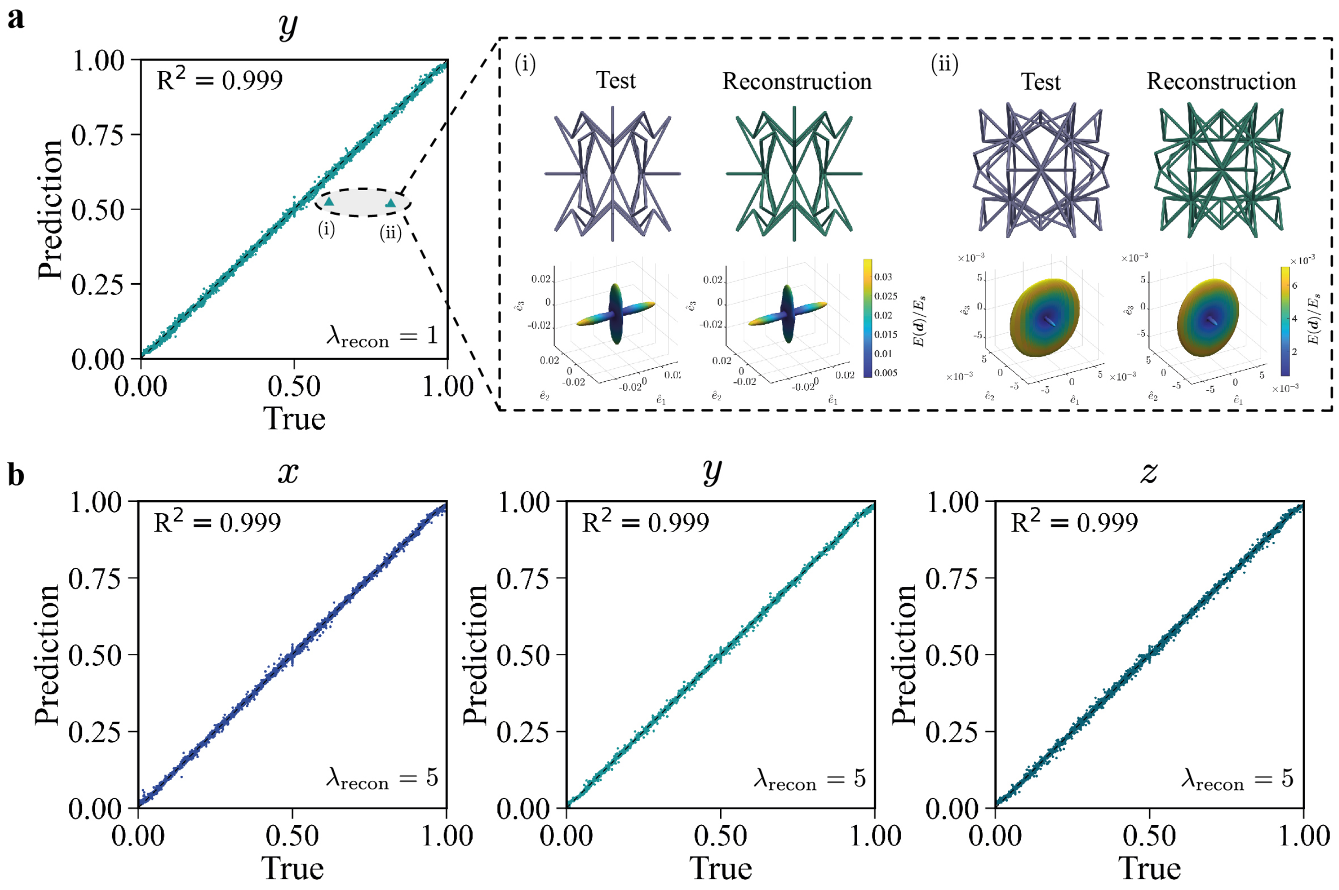

Supplementary Figure 8. Evaluation of the reconstruction accuracy of the VAE model. **(a)** Illustration of two selected extreme outliers from Supplementary Fig. 6a, showing the truss structures and their corresponding elastic surfaces. **(b)** Reconstructed vs. true 3D components (x, y, z) of the node positions in the test dataset using $\lambda_{\text{recon}} = 5$. Dashed lines represent the ideal lines with zero-intercept and unit-slope; the corresponding coefficient of determination R^2-scores are indicated.

the same test dataset used in the manuscript), which, however, can lead to a compromised or degraded property predictor accuracy due to the finite total capacity of the model, as shown in Supplementary Table 3.

R^2-scores	x	y	z	$\mathbb{C}_{1111}$	$\mathbb{C}_{1122}$	$\mathbb{C}_{1133}$	$\mathbb{C}_{2222}$	$\mathbb{C}_{2233}$	$\mathbb{C}_{3333}$	$\mathbb{C}_{2323}$	$\mathbb{C}_{3131}$	$\mathbb{C}_{1212}$
$\lambda_{\text{recon}} = 1$	0.999	0.999	0.999	0.995	0.987	0.989	0.995	0.990	0.995	0.983	0.983	0.982
$\lambda_{\text{recon}} = 5$	0.999	0.999	0.999	0.982	0.961	0.966	0.981	0.965	0.979	0.980	0.981	0.976

Supplementary Table 3. Comparison of the node positions' reconstruction accuracy of the VAE model and the prediction accuracy of the property predictor, using $\lambda_{\text{recon}} = 1$ and $\lambda_{\text{recon}} = 5$.

3 Exploration in the latent space

3.1 Sampling in the latent space

To illustrate that the VAE model tends to encode lattices with similar topologies and mechanical properties in the same region of the latent space, we randomly picked a starting point $\boldsymbol{z}_0$ in the latent space and sample points in its vicinity by adding Gaussian noise to the point, according to

$$\boldsymbol{z} = \boldsymbol{z}_0 + \beta \cdot \boldsymbol{\varepsilon}, \qquad \text{with} \quad \boldsymbol{\varepsilon} \sim \mathcal{N}(\boldsymbol{0}, \boldsymbol{I}), \tag{5}$$

where $\beta \in [0, 1]$ is a scaling factor that determines the range of sampling. Supplementary Figure 9c shows representative examples of structures obtained by decoding the newly sampled points $\boldsymbol{z}$, along with their corresponding elastic surfaces computed by FEM homogenization. We observe that randomly sampled points in the neighborhood of the starting point exhibit similar topological features and mechanical properties. This similarity can be attributed to the property predictor that aims to learn the mapping from the latent space to properties. By incorporating physical knowledge into the VAE model, the predictor encourages the model to generate similar latent representations for trusses with similar properties for better prediction performance, as demonstrated in previous work with a VAE setup[9] (albeit in a completely different context).

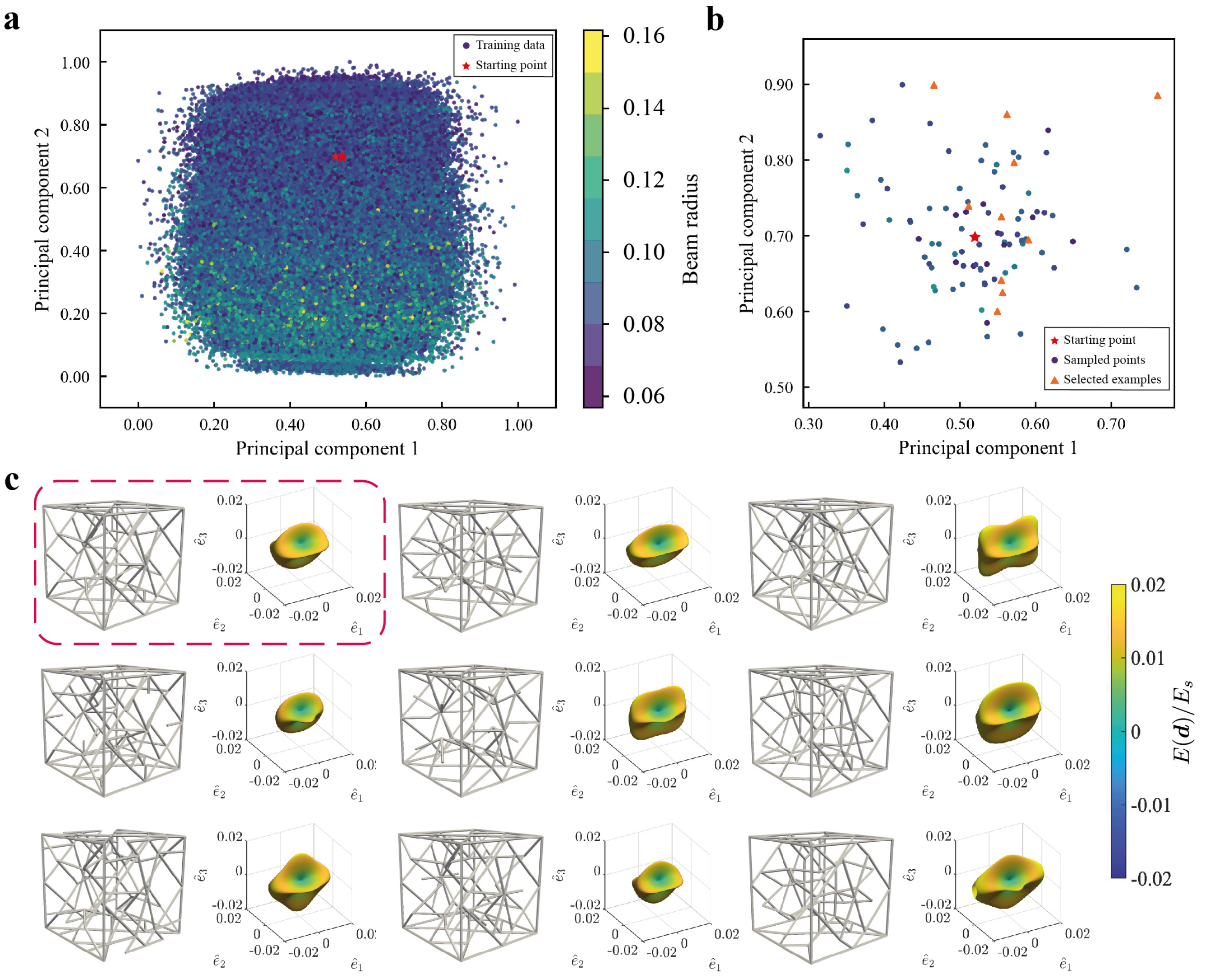

Supplementary Figure 9. Representations of sampling results in the latent space. **(a)** Two-dimensional PCA analysis of the latent space of the VAE model jointly trained with the property predictor. Colors indicate the beam radius of the truss structure. **(b)** Illustration of points sampled in the vicinity of the starting point ($\beta = 0.05$). **(c)** Representative examples of truss structures and their corresponding elastic surface generated by decoding from randomly-sampled points from the latent space. Highlighted in red in (a) and (b) is the starting point $\boldsymbol{z}_0$.

3.2 Interpolation in the latent space

Figure 3 of the main article shows representative examples of interpolation in the latent space. Specifically, the truss structures presented in Figure 3a were generated by traversals along different latent axes, which involves randomly selecting a truss structure, mapping it to the latent space to obtain its latent representation, and independently modifying each of the latent variables (corresponding to topology-specific, geometry-specific, and shared dimensions

in the latent space) along their negative and positive directions with a fixed step size. When interpolating between two points whose corresponding trusses exhibit extreme mechanical properties (as illustrated in Figure 3b of the manuscript), we employ *spherical linear* interpolation (slerp) rather than linear interpolation (lerp) in the latent space.

The choice of slerp over lerp is motivated by several factors. Constructing an approximately continuous latent space of truss structures provides a significant advantage, as the vector representation of truss lattices allows for the generation of novel structures by arithmetic operations. However, performing interpolation in a high-dimensional latent space with a Gaussian prior presents challenges. First, lerp assumes a straight line between points, which ignores the underlying structure of the data distribution, as schematically shown in Supplementary Fig. 10a below. Additionally, lerp measures the Euclidean distance between two points, which, however, does not necessarily indicate the similarity between truss structures in high-dimensional spaces. Consequently, the linearly interpolated points may jump or traverse regions of the latent space with similar representations, leading to inconsistent or unrealistic interpolations. In contrast, slerp takes into account the spherical structure of the latent space and follows the shortest arc on a hypersphere, which reduces artifacts or unnatural transitions between points. Supplementary Figure 11 shows that linear interpolation in our truss latent space produces less coherent geometries with abrupt changes in properties, whereas slerp interpolation (as shown in Figure 3b in the main article) exhibits smoother transitions in both geometries and properties along the interpolation path. The effectiveness of slerp has been demonstrated in the context of various generative models[9,10] with uniform and Gaussian priors. Overall, slerp is generally more suitable for generating smooth and meaningful interpolations in the latent space of generative models.

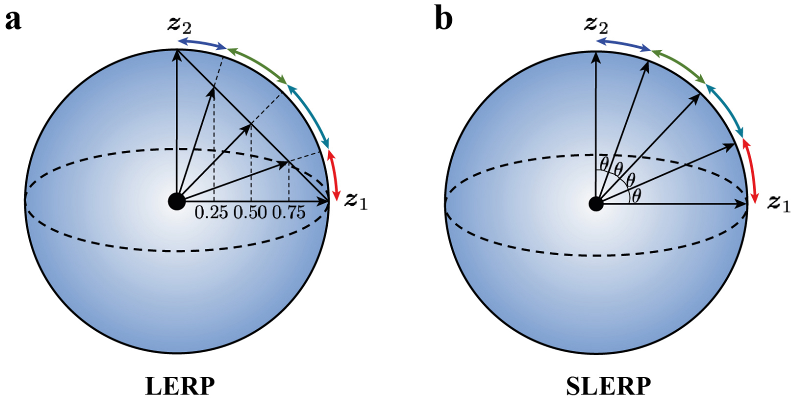

Supplementary Figure 10. Illustration of the lerp and slerp interpolations with interpolation parameter $\alpha \in \{0.25, 0.5, 0.75\}$. (a) *Linear* interpolation: interpolated vectors are obtained by $\text{LERP}(\boldsymbol{z}_1, \boldsymbol{z}_2; \alpha) = \alpha \cdot \boldsymbol{z}_1 + (1-\alpha) \cdot \boldsymbol{z}_2$. **(b)** *Spherical* interpolation: the interpolation is based on the angle between two points, and the resulting interpolation path follows a great circle on the hypersphere.

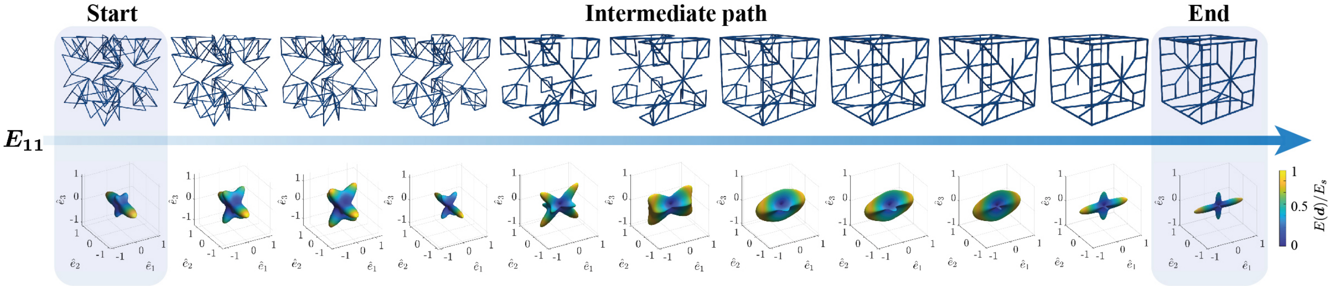

Supplementary Figure 11. Representative examples of linear (lerp) interpolation in the latent space. Samples are obtained by linear interpolation between two points in the latent space, whose corresponding trusses exhibit extreme directional Young's modulus E_{11}. Their corresponding 3D elastic surface evolution (obtained by finite element homogenization) is shown along the interpolation path.

3.3 Gradient-based optimization in the latent space

For each optimization task, we first evaluate structures in the training dataset and select the 100 closest matches in terms of the target property as the initial guesses. Then, we perform gradient-based optimization for each initial guess in parallel and identify the optimal solution followed by FE homogenization-based validation of their properties. As a representative example, Supplementary Figure 12a illustrates truss structures obtained along the optimization path of minimizing Poisson's ratio ν_{21} (as shown in Figure 4b in the main article), which showcases the evolution of the geometries and properties.

To further emphasize that multiple similar truss structures can be generated, we have sampled in the neighborhood of the optimal solution based on Supplementary Equation 5. Supplementary Figure 12b shows that novel structures generated by sampling in the neighborhood of a point in the latent space exhibit similar topological features and mechanical properties (see also Supplementary Fig. 9). Notably, some structures generated through sampling in the latent space even display superior target properties compared with the solution we obtained through optimization, which highlights the potential of our generative framework in not only memorizing the training data but generalizing from a comprehensive truss dataset, effectively capturing the underlying mechanical relations. This finding further reinforces the notion that our generative framework is highly structured and is capable of leveraging the encoded mechanical property information within the latent space.

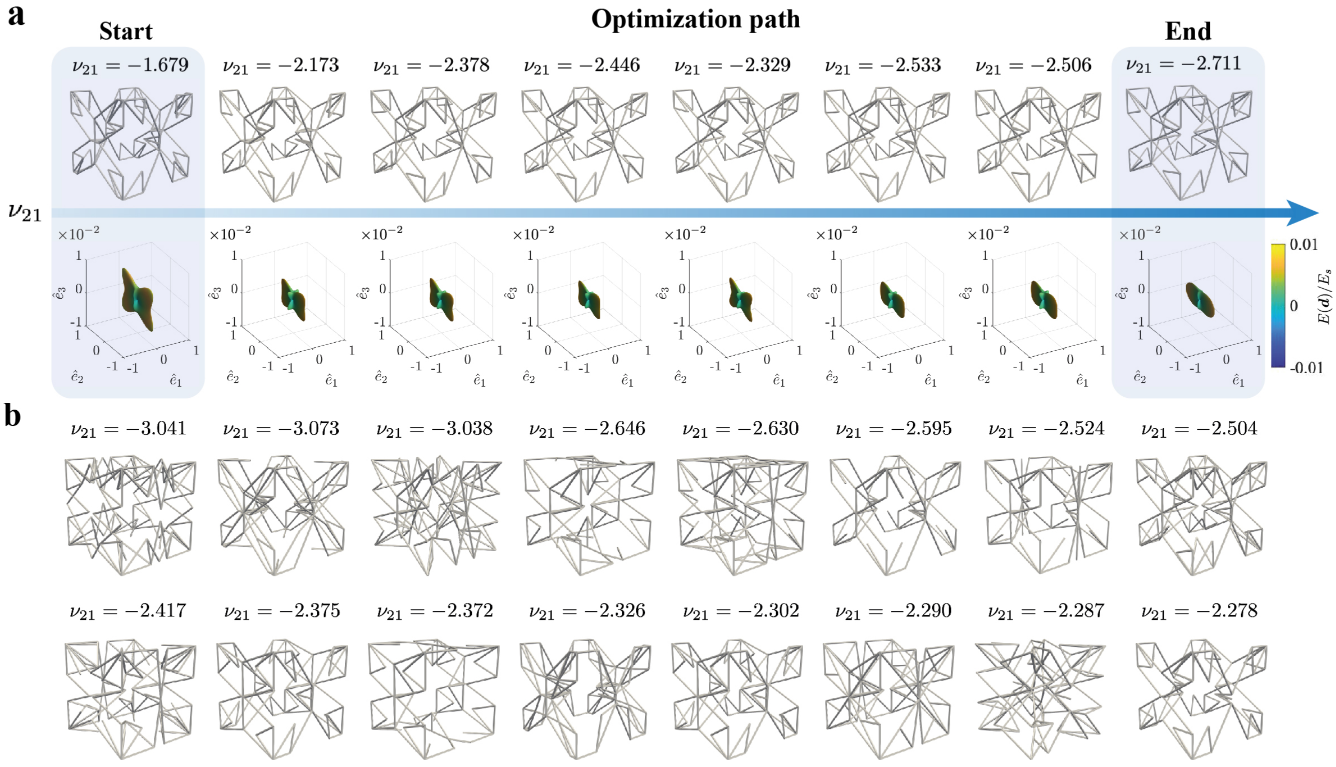

Supplementary Figure 12. Truss structures obtained from gradient optimization to minimize Poisson's ratio ν_{21}. (a) Evolution of truss geometries and their elastic stiffness properties along the optimization path. **(b)** Representative examples of truss structures obtained by sampling in the vicinity of the optimal solution in (a) with the sampling factor $\beta = 0.05$ using Supplementary Equation 5. The shown elastic stiffness surfaces were obtained by finite element homogenization.

3.4 Inverse design of truss metamaterials with target nonlinear responses

To characterize the effective nonlinear behavior of periodic trusses, we compute the 3D stress-strain responses of truss unit cells under uniaxial compression. Relying on the principles of computational homogenization, we impose average strain states through periodic boundary conditions, leveraging the FE framework implemented in the open-source code AE108 (available at `http://ae108.ethz.ch`). Considering linear elastic Timoshenko beam elements to represent each strut in the truss unit cell, a compressive strain of up to 25% is applied to the unit cell in increments. The unit cell is connected to two rigid plates at the top and bottom surfaces, with a displacement boundary condition

applied at the top surface in the negative z-direction and a fixed bottom surface. Periodic boundary conditions are applied to the displacements on the remaining four surfaces (and to rotations on all surfaces), thus mimicking the response of an infinite periodic sheet of truss unit cells. The corresponding nodal reaction forces at the top (or bottom) face are used to extract the effective compressive stress.

To enhance the training efficiency without sacrificing accuracy, we reduce the dimensionality of the learning labels by selecting representative points along the range of applied loads and reconstructing the full response by interpolation during postprocessing. The reduced dimension of labels is determined by hyperparameter tuning to ensure the representation capacity of the model. To this end, we describe each truss structure as $(\boldsymbol{A}, \boldsymbol{x})$, where $\boldsymbol{A}$ and $\boldsymbol{x}$ represent the connectivity matrix and the node offsets (as before), and the corresponding stress-strain curve as a 13-dimensional vector $\boldsymbol{\sigma}_t = [\sigma(0.5\%), \sigma(2.5\%), \cdots, \sigma(24.5\%)]^T$, which contains the compressive stress values at the 13 equally spaced strain values $\varepsilon = 0.5\%, 2.5\%, \ldots, 24.5\%$. In this task, we construct a dataset that comprises 383,729 unique structures using the same parameterization as described in the manuscript, and their corresponding stress-strain responses for training the generative modeling framework towards the inverse design of truss structures with target nonlinear behaviors. The property predictor is trained to learn the mapping from the low-dimensional latent representations to the nonlinear stress-strain responses, which is then integrated into a gradient-based optimization framework (details described in Supplementary Note 3.3) to search for optimal designs that exhibit desired nonlinear behaviors.

4 Computational efficiency estimates

To demonstrate the efficiency of our approach, Supplementary Table 4 presents an overview of the computational runtime, the software and hardware resources required for different tasks. Once trained offline, the property predictor can provide accurate stiffness predictions instantly. The computational time for stiffness calculation is significantly reduced compared to FEM homogenization, which provides a convenient shortcut for the optimal design of truss lattices.

Tasks	Software	Hardware	Runtimes
Dataset generation	Python	CPU (30 cores)†	4 hours
Stiffness computations (of the full dataset) using FEM	In-house C++ FEM code	CPU (30 cores)†	20 hours
Joint training of the VAE and property predictor $\mathcal{F}_\omega$	PyTorch in Python	GPU§	4 hours
Stiffness prediction (of 2000 trusses) using $\mathcal{F}_\omega$ ¶	PyTorch in Python	GPU§	0.001 seconds
Optimal truss design using gradient-based optimization *	PyTorch in Python	CPU (30 cores)†	5 minutes

Supplementary Table 4. Overview of the computational runtime, the software and hardware resources required for different tasks. The reported runtimes are rough average estimates. †Computations were performed on the Euler IV cluster of ETH Zurich with two 18-core 2.7 GHz Intel Xeon Gold 6150 processors and 192 GB of DDR4 memory at 2666 MHz. §Computations were performed on a single Nvidia Quadro RTX 6000 24 GB GDDR6. ¶Runtimes for the property predictor are measured for one prediction on 2000 data samples. *Runtimes for optimization via the proposed ML framework are reported for parallel computation with 100 initial guesses (averaged over the different tasks shown in the main article).

Supplementary References

1. Panetta, J. *et al.* Elastic textures for additive fabrication. *ACM Transactions on Graph. (TOG)* **34** (2015).

2. Maurizi, M., Gao, C. & Berto, F. Inverse design of truss lattice materials with superior buckling resistance. *npj Comput. Mater.* **8**, 247 (2022).

3. Wang, Y., Zeng, Q., Wang, J., Li, Y. & Fang, D. Inverse design of shell-based mechanical metamaterial with customized loading curves based on machine learning and genetic algorithm. *Comput. Methods Appl. Mech. Eng.* **401**, 115571 (2022).

4. Deng, B. *et al.* Inverse design of mechanical metamaterials with target nonlinear response via a neural accelerated evolution strategy. *Adv. Mater.* **34**, 2206238 (2022).

5. Kingma, D. P. & Ba, J. Adam: A method for stochastic optimization. *arXiv preprint arXiv:1412.6980* (2014).

6. Lerique, S., Abitbol, J. L. & Karsai, M. Joint embedding of structure and features via graph convolutional networks. *Appl. Netw. Sci.* **5**, 1–24 (2020).

7. Lipton, Z. C. The mythos of model interpretability: In machine learning, the concept of interpretability is both important and slippery. *Queue* **16**, 31–57 (2018).

8. Montero, M. L., Ludwig, C. J., Costa, R. P., Malhotra, G. & Bowers, J. The role of disentanglement in generalisation. In *International Conference on Learning Representations* (2021).

9. Gómez-Bombarelli, R. *et al.* Automatic chemical design using a data-driven continuous representation of molecules. *ACS Cent. Sci.* **4**, 268–276 (2018).

10. White, T. Sampling generative networks. *arXiv preprint arXiv:1609.04468* (2016).
