# Supplementary material for: Unifying the design space and optimizing linear and nonlinear truss metamaterials by generative modeling
Source: Nat Commun. 2023 Nov 21;14:7563. doi: 10.1038/s41467-023-42068-x (PMC10663604; doi:10.1038/s41467-023-42068-x)
Supplement: Supplementary file 3 — Description of Additional Supplementary Files [file 41467_2023_42068_MOESM3_ESM.pdf]

<u>Description of Additional Supplementary Files</u>

Supplementary Movie 1: Representative examples of interpolation in the latent space (latent axis 1).

Supplementary Movie 2: Representative examples of interpolation in the latent space (latent axis 2).

Supplementary Movie 3: Representative examples of interpolation in the latent space (latent axis 3).

Supplementary Movie 4: Representative examples of interpolation in the latent space (in terms of E_{11}).

Supplementary Movie 5: Representative examples of interpolation in the latent space (in terms of A^U).
